# Opsin expression patterns coincide with photoreceptor development during pupal development in the honey bee, *Apis mellifera*

**DOI:** 10.1186/s12861-018-0162-8

**Published:** 2018-01-30

**Authors:** Leonie Lichtenstein, Kornelia Grübel, Johannes Spaethe

**Affiliations:** Department of Behavioral Physiology and Sociobiology, Biozentrum, University of Würzburg, Würzburg, Germany

**Keywords:** Insect vision, Photoreceptor, Spectral sensitivity, Visual pigments, Behavioral transition

## Abstract

**Background:**

The compound eyes of insects allow them to catch photons and convert the energy into electric signals. All compound eyes consist of numerous ommatidia, each comprising a fixed number of photoreceptors. Different ommatidial types are characterized by a specific set of photoreceptors differing in spectral sensitivity. In honey bees, males and females possess different ommatidial types forming distinct retinal mosaics. However, data are lacking on retinal ontogeny and the mechanisms by which the eyes are patterned. In this study, we investigated the intrinsic temporal and circadian expression patterns of the opsins that give rise to the ultraviolet, blue and green sensitive photoreceptors, as well as the morphological maturation of the retina during pupal development of honey bees.

**Results:**

qPCR and histological labeling revealed that temporal opsin mRNA expression differs between sexes and correlates with rhabdom elongation during photoreceptor development. In the first half of the pupal stage, when the rhabdoms of the photoreceptors are still short, worker and (dorsal) drone retinae exhibit similar expression patterns with relatively high levels of UV (*UVop*) and only marginal levels of blue (*BLop*) and green (*Lop1*) opsin mRNA. In the second half of pupation, when photoreceptors and rhabdoms elongate, opsin expression in workers becomes dominated by *Lop1* mRNA. In contrast, the dorsal drone eye shows high expression levels of *UVop* and *BLop* mRNA, whereas *Lop1* mRNA level decreases. Interestingly, opsin expression levels increase up to 22-fold during early adult life. We also found evidence that opsin expression in adult bees is under the control of the endogenous clock.

**Conclusions:**

Our data indicate that the formation of the sex-specific retinal composition of photoreceptors takes place during the second half of the pupal development, and that opsin mRNA expression levels continue to increase in young bees, which stands in contrast to *Drosophila*, where the highest expression levels are found during the late pupal stage and remain constant in adults. From an evolutionary perspective, we hypothesize that the delayed retinal maturation during the early adult phase is linked to the delayed transition from indoor to outdoor activities in bees, when vision becomes important.

## Background

Detecting and processing light is crucial for the survival and reproduction of nearly all animals. Aside from various forms of vision, the perception of light is important for entraining the endogenous clock. Insects represent one of the most diverse groups of the animal kingdom and have colonized almost all habitats; they usually possess a pair of compound eyes that converts photon energy into an electric signal for further processing [1, 2]. The retinae of all compound eyes are composed of repetitive functional units, termed ommatidia. In turn, each ommatidium consists of a dioptric system which includes the cornea and the crystalline cone cells forming the lens system, and a specialized receptor system comprising a fixed number of photoreceptor cells. Depending on the species, photoreceptors form open or fused light-guiding structures, the rhabdomeres and rhabdoms, respectively. These structures contain the photopigments which are essential for phototransduction [3–5]. Each photoreceptor cell expresses a specific visual pigment, a protein of the G-coupled opsin family, and its conjugated chromophore, the retinal [6]. Although the chromophore absorbs photons and changes its conformation, the spectral sensitivity of a photoreceptor is mainly defined by the interaction between the chromophore and its respective opsin protein [7–9].

In a wide range of animal species, it has been shown that different photoreceptor types, which are characterized by a distinct spectral sensitivity and a related opsin protein, form random mosaics, localized zones or bands within the retina [2, 10]. In the last decade, much effort has been devoted to understanding the molecular and developmental mechanisms giving rise to different photoreceptor types and species-specific patterns, as well as sex-specific patterns of retinal mosaics [11, 12].

Retinal mosaics and distinct photoreceptor distributions are suggested to be present in the compound eyes of workers and drones of the honey bee (*Apis mellifera*), which is recognized as an insect model for visual (color) perception, learning and memory [13]. Interestingly, the development of the honey bee retina including photoreceptors and rhabdoms, initiates in the late larval stage and reaches its final form in the late pupal phase [14–16]. By means of electrophysiological recordings of single photoreceptor cells and by molecular studies, three spectral photoreceptor types have been identified in honey bees, that are most sensitive in the ultraviolet (UV, ca. 340 nm), blue (ca. 430 nm) and green (ca. 540 nm) part of the light spectrum [17–20]. Moreover, these three photoreceptor types give rise to at least three types of ommatidia in worker eyes, that comprise different sets of photoreceptors. Each type contains six green photoreceptor cells, and additionally either one UV and one blue photoreceptor (type I), two UV photoreceptors (type II), or two blue photoreceptors (type III), which are more or less randomly distributed in the main retina (the sensitivity of the short distal cell is unknown) [21]. In contrast, compound eyes of drones are divided into a male-specific dorsal part, which consists mainly of UV and blue photoreceptor cells, and a small remaining ventral part, which shows a photoreceptor composition that is similar to the compound eye of workers [17, 22, 23]. Despite the existence of various spectral ommatidial types and a sex-specific ommatidial distribution in the honey bee retina, it is neither known which developmental and molecular mechanisms underlie the distinct arrangement of photoreceptors, nor when different opsins are going to be expressed during retinal development.

In this study we aim to contribute to a better understand of retinal development and photoreceptor determination in honey bees. Thus, we first investigated the intrinsic temporal onset of opsin expression patterns during pupal and adolescent development of the honey bee worker and drone compound eye by quantifying opsin mRNA expression in the retina. Second, we used confocal microscopy to characterize the morphological development of the retina with special focus on the light-guiding fused rhabdoms, in which the visual pigments are embedded, to screen for possible correlations between morphological and molecular changes. Third, we also tested whether the opsin expression in young adult bees is under circadian control since this has been shown in other animals, e.g. Noctuidae [24] and vertebrates [25].

## Results

### Temporal opsin expression patterns of honey bee workers and drones

**Fig. 1:**
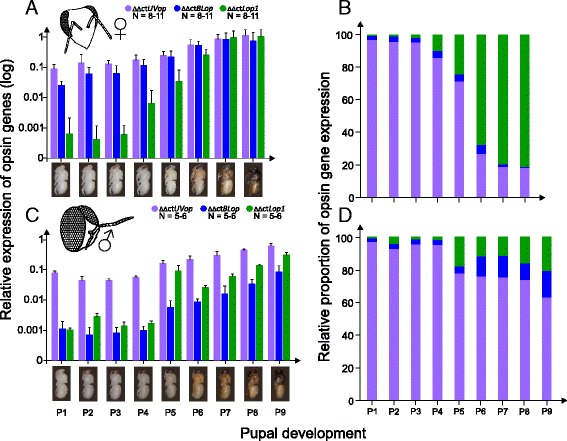
Temporal opsin expression during pupal development of workers and drones. **a**,**c**: Relative mRNA expression levels of the ultraviolet (*UVop*), blue (*BLop*) and green (*Lop1*) opsin in the compound eyes during pupal development of workers (**a**; pupal stages: P1-P8) and drones (**c**; P1-P9; only the dorsal part of the compound eye: grey area of the retina marked in the inset; note that the pupal phase of drones is one day longer than in workers) by means of qPCR. Expression level for each opsin gene was normalized to the reference gene *Rp49*, and the level of newly emerged bees was set to one (NE; see Fig. 2). **b**,**d**: Relative proportion of opsin mRNA expression in workers (**b**) and drones (**d**) at different pupal stages. For calculation, see [55]. Error bars indicate standard deviation

**Fig. 2:**
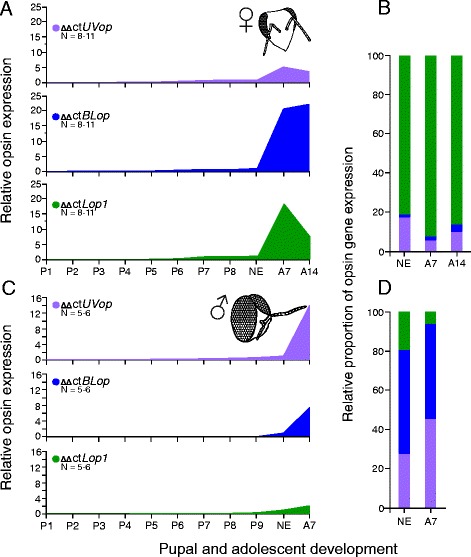
Overview of opsin expression during pupal and adolescent development of workers and drones. Relative mRNA expression levels of ultraviolet (*UVop)*, blue (*BLop*) and green (*Lop1*) opsin genes in the compound eyes were evaluated during pupal and early adult development of workers (upper half) and drones (lower half; only the dorsal part of the compound eye) by means of qPCR. Opsin expression was normalized separately for each opsin gene to the expression level of newly emerged bees. **a**,**c**: Opsin mRNA levels in both sexes strongly increased after eclosion up to 22-fold. **b**,**d**: In contrast to the pupal phase, the relative proportions of opsin expression levels remained constant after eclosion in workers but not in drones. Error bars indicate standard deviation. NE, newly emerged; A7, 7-day old; A14, 14-day old

In both sexes, *UVop* mRNA was found to be expressed during the first pupal stage and *Lop1* mRNA at about mid-pupation. In contrast, *BLop* mRNA expression commenced in drones much later than in workers (Fig. 1). Overall, the mRNA expression levels of *UVop*, *BLop* and *Lop1* mRNA significantly increased during pupal development (GLM, Poisson family, *P* < 0.001; *UVop*: ${\chi}_8^2$ = 2,361,600; *BLop*: ${\chi}_8^2$ = 2,117,777; *Lop1*:${\chi}_8^2$ = 4,084,108) and differed significantly between workers and drones (GLM, Poisson family, *P* < 0.001; *UVop*: ${\chi}_1^2$= 1,042,426; *BLop*: ${\chi}_1^2$ = 1,798,472; *Lop1*:${\chi}_1^2$ = 1,320,809; Fig. 1a and c). Furthermore, we found a significant interaction between pupal stage and sex (GLM, Poisson family, *P* < 0.001; *UVop*:${\chi}_7^2$ = 20,020; *BLop*: ${\chi}_7^2$ = 8449; *Lop1*: ${\chi}_7^2$ = 73,288). This suggests that different mechanisms underlie drone and worker retinal development. Comparing the relative proportions of opsin expression between sexes shows that in workers and drones, *UVop* mRNA is already expressed during the first pupal stage. In the second half of pupal development, *Lop1* mRNA expression strongly increases in workers, whereas in the dorsal part of the drone eye *Lop1* mRNA increases only slightly compared to *UVop* and *BLop* mRNA (Figs. 1 and 2). However, in both workers and drones, opsin expression levels strongly increased after eclosion up to 22-fold, even though all bees were kept in constant darkness (Fig. 2). In workers, the relative proportions of opsin expression levels remained rather constant after eclosion, whereas young drones showed a strong increase in *UVop* and *BLop* mRNA expression levels, while the relative *Lop1* mRNA expression level dropped during adolescent development (Fig. 2d).

### Circadian opsin expression patterns of honey bee workers

**Fig. 3:**
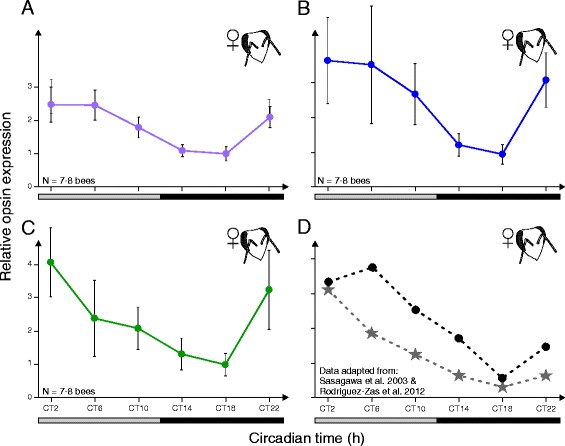
Circadian opsin expression patterns of workers. **a**,**b**,**c**: Relative mRNA expression levels of ultraviolet (*UVop*; **a**), blue (*BLop*; **b**) and green (*Lop1*; **c**) opsin genes in the compound eyes of 14-day old workers at six different circadian time points (CT2-CT22). Expression level for each opsin gene was normalized to the reference gene *Rp49* and the lowest expression level was set to one. Expression levels were highest shortly after the expected light onset (CT2-CT6) and lowest in the middle of the expected night (CT18). **d**: Expression data of *Lop1* compiled from earlier studies for comparison (asterisk: [26], black dots: [27]). Error bars indicate standard deviation

In 14-day old bees, which were entrained for 13 days under a light/dark cycle but kept under constant darkness on day 14, we found that *UVop (*ANOVA*: P <* 0.01; *F*_*6,14*_ = 3.5239), *BLop (*ANOVA: *P* = 0.05; *F*_*6,14*_ = 2.3239) and *Lop1* (ANOVA: *P <* 0.05; *F*_*6,14*_ = 2.5316) exhibited a similar oscillation pattern for 24 h (Fig. 3). For all opsins, mRNA expression was the highest shortly after the expected light onset at CT2 – CT6 and the lowest in the middle of the expected night at CT18 (Fig. 3). This pattern matches the circadian expression pattern of the *Lop1* in workers described in earlier studies by means of northern-blotting and qPCR (Fig. 3d; [26, 27]).

### Retinal development in the honey bee worker

**Fig. 4:**
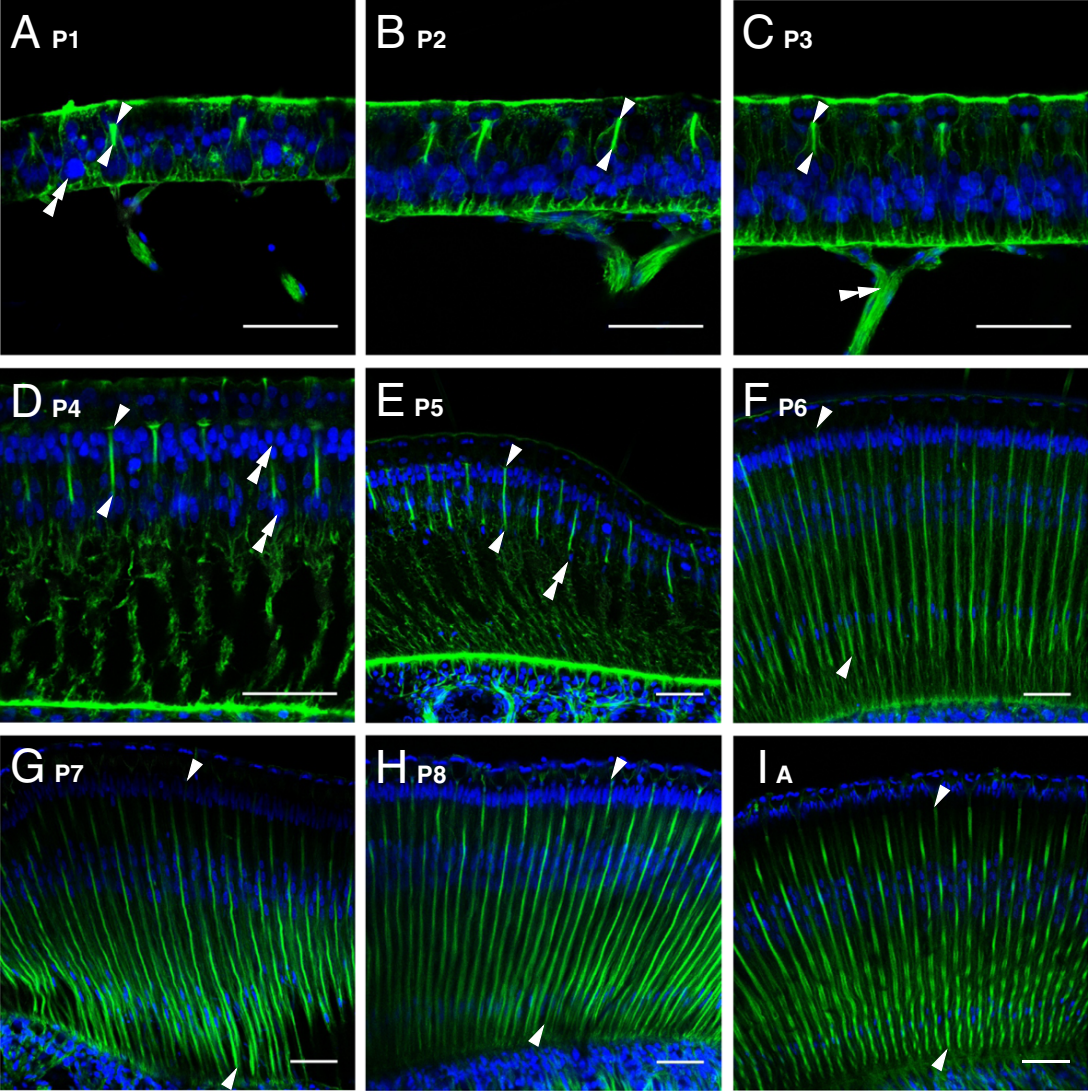
Retinal development in the worker pupae. Histological labeling of frontal sections at approximately the center of the eye with phalloidin (green) and Hoechst 34580 (blue) to label f-actin and nuclei, respectively, were performed to visualize the retinal development of the honey bee compound eyes. **a**-**c**: At the beginning of the pupal phase the retina consists only of a thin hypodermis comprising crystalline cone cells, several pigment cells, hair cells (double arrow head in **a**) and photoreceptor cells which contain the fused rhabdoms (distal and proximal boundaries of the rhabdom are marked by arrow heads) and extend their axons as twisted bundles (double arrow head in **c**) through the basement membrane. At this early pupal phase rhabdoms are short and all nuclei (except those from the crystalline cone cells) are clustered in the same level. **d**-**h**: During the next days of pupal development rhabdoms and photoreceptor cells significantly increase in size, and nuclei of photoreceptor and pigment cells separate from each other into distinct layers (double arrow head in **d**). The cell nuclei of the ninth photoreceptor cell become first visible in pupal stage P5 and form a distinct layer in P6 (double arrow head in **e**). **i**: The retina reached its final stage in freshly eclosed bees (A, adult). Scale bar in all figures: 50 μm

**Fig. 5:**
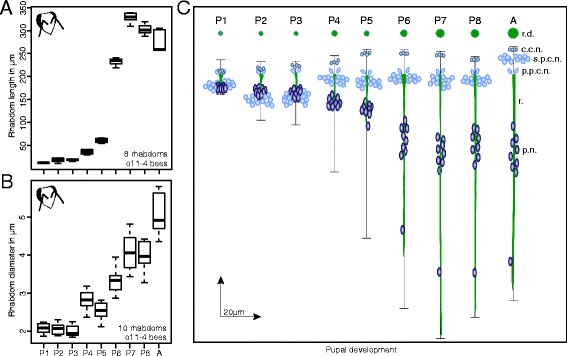
Rhabdom and photoreceptor development in the worker. **a**: Rhabdom length and **b**: rhabdom diameter shown for all pupal stages and freshly eclosed bees. **c**: Overview of rhabdom (green bar) and photoreceptor cell elongation (black line), increase in rhabdom diameter (r.d.) and segregation of nuclei from photoreceptor, pigment and crystalline cone cells. c.c.n.: crystalline cone nuclei; s.p.c.n.: secondary pigment cell nuclei; p.p.c.n.: primary pigment cell nuclei; r.: rhabdom; p.n.: photoreceptor nuclei

To evaluate modification of the retina and rhabdoms during pupal development, we performed histological labeling of the retina at all pupal stages and in adult worker bees, and quantified rhabdom length and diameter by means of the phalloidin (f-actin) imaging data. Rhabdom length and diameter significantly increased during pupal development from ca. 13 μm length in 1-day old pupae to ca. 300 μm in adult bees, and from ca. 2 μm in diameter in 1-day old pupae to ca. 5 μm in adult bees (Fig. 5; Kruskal-Wallis-Test, length: *P <* 0.001; ${\chi}_8^2$= 68.658; diameter: *P <* 0.001; ${\chi}_{10}^2$= 81,679). During the first pupal stage (Fig. 4a), the retina consisted only of a thin hypodermis, and each ommatidium comprised four crystalline cone cells, as well as several pigment and photoreceptor cells, which extended their axons through the basement membrane towards the first optical ganglion, the lamina (see also: [14, 28]). However, except for the four crystalline cone nuclei, all other nuclei of the photoreceptor and pigment cells were clustered in the same level. At this early phase (Fig. 4a; P1), the rhabdoms were short (~ 13 μm) and occurred only in the apical part of photoreceptors, just beneath the crystalline cones. During the next few days, rhabdom length increased significantly, with a large step in the middle of the pupal phase between P5 to P6, where the length almost quadrupled from ca. 60 μm to 232 μm (Fig. 4e and f). Shortly before eclosion, the rhabdoms stretched through the whole photoreceptors. Aside from rhabdom growth, photoreceptors increased in size, and the nuclei of photoreceptors and pigment cells separated from each other into two distinct layers (Fig. 4d). In a later stage, the nuclei of photoreceptors and pigment cells further segregated from each other (Fig. 4d-i), and the cell nucleus of the ninth photoreceptor cell became visible (Fig. 4e and f). During the final phase of pupation, rhabdoms became more compressed which thus led to a slight reduction in total rhabdom length (Fig. 5c). In addition to an increase in length, rhabdom diameter significantly increased about threefold between P3 and the adult stage (Fig. 5a and b).

## Discussion

In the present study, we showed that three opsin genes, *UVop*, *BLop* and *Lop1* are expressed in the retina during pupal development of the honey bee. Opsin expression patterns differ between sexes, and the onset of UV, blue and green opsin mRNA occurs at different times during development (Figs. 1 and 2). At early pupal stages, worker and (dorsal) drone compound eyes exhibit similar expression levels with relatively high levels of *UVop* and only marginal levels of *BLop* and *Lop1* mRNA. This expression pattern significantly changes at about mid-pupation (Fig. 1). Opsin expression in workers becomes dominated by *Lop1* mRNA (Fig. 1a and b), which corresponds to the high number of green photoreceptor cells (six out of eight large PR cells) in the worker eye, as revealed by in situ hybridization [21]. In contrast, the dorsal drone eye shows high expression levels of *UVop* and *BLop* mRNA, whereas *Lop1* mRNA level decreases (Fig. 1c and d), which approximates the proportion of UV and blue-sensitive photoreceptors that is found in the dorsal part of the drone eye ([17, 22, 23]; Fig. 1c and d). Surprisingly, in both workers and drones *UVop* mRNA is expressed during the first pupal stage (P1; Fig. 1) and at least in workers we confirmed that *UVop* mRNA is also present in late larval stages (unpublished data). Larval and pupal development of the Western honey bee takes place inside the nest under constant darkness [29] and thus the functional significance of an early (UV) opsin expression is unclear. UV opsin expression with an unknown function has been described in the antennal lobes of adult bumblebees [30]. Furthermore, arrestin, which interacts with rhodopsin and is essential in visual processing, has also been detected in antennal lobes of flies where it is required for normal olfactory physiology in a non-visual process [31]. In *Drosophila*, *Rh1* opsin gene expression is essential for correct rhabdomere development [32], and similar mechanisms might also exist in the honey bee.

*BLop* mRNA expression commences early in workers (P1-2) but much later in drones (P5). In contrast, *Lop1* mRNA expression starts approximately at mid-pupation (P4-5) in both sexes. Interestingly, physiological studies have found no green-sensitive photoreceptors in the dorsal eye of adult drones [17, 22], whereas our study demonstrates that up to 20% of *Lop1* mRNA is expressed by the end of the pupal phase. Since we observed only low *Lop1* mRNA expression levels during pupation, and the relative proportion of *Lop1* mRNA expression obviously decreases during adolescent development, regulatory post-transcriptional processes [33] could lead to a degeneration of the *Lop1* mRNA or protein in adult drones, which may explain the lack of green photoreceptor cells in the dorsal part of the adult drone eye. Alternatively, *Lop1* mRNA is only transiently expressed in dorsal drone eyes and never translated into protein, as recently been suggested for the PxG2 opsin in some photoreceptors of the type I and II ommatidia during retinal development of the butterfly *Papilio xuthus* [34]. Since intracellular recording from individual photoreceptors is difficult, this approach may have missed very rare photoreceptor types [17]. Thus, future studies using specific antibodies against the Lop1 opsin are needed to verify the presence (or absence) of green-sensitive photoreceptors in the dorsal part of the drone compound eye.

Visual pigments are located in the fused rhabdom of the bees’ photoreceptor cells, which functions as a light guide to convey the photons from the periphery to the light-sensitive molecules [5, 6]. During pupal development, we found a slight but steady increase of opsin expression levels, which goes hand in hand with the morphological differentiation of the rhabdoms (Figs. 4 and 5). Since Phillips [14] first published details on the development of the honey bee retina in the early twentieth century, surprisingly few studies have investigated the development of the photoreceptors in the bee compound eye. The formation of the retina starts during the larval phase, and the photoreceptors within the ommatidia are fully differentiated and arranged by the end of the pre-pupal phase (Philips 1905). In addition, the optic lobes gradually start to develop their final form during the last larval stage when optic nerve projections reach the lamina [35]. During pupal development, the ommatidia lengthen and cellular elements are fully differentiated by the time when all ommatidia have reached their final adult shape [14]. Rhabdom development starts during the end of the larval phase and is characterized by the formation of a cavity along the ommatidial axis to which rhabdomeres could extend by invagination of the plasma membrane [15]. At the beginning of the pupal phase, and in congruence with our observations, rhabdoms are very short and therefore are only present in the apical part just underneath the crystalline cone cells (Fig. 4a). However, rhabdoms lengthen rapidly during pupal development until they reach the distal part of the basement membrane [14, 15]. We observed the most obvious leap in rhabdom extension and photoreceptor cell differentiation at mid-pupation (P5), which coincides with the first distinct increase of opsin expression levels of all three investigated opsins (Figs. 1 and 5). Overall, our results clearly show that ommatidial development and opsin expression during the pupal phase differs between the honey bee and *Drosophila*. Whereas in honey bees, opsin expression and rhabdom maturation co-occur, opsin expression in flies is initiated much later near the end of the retina completion [32, 36].

Based on our results, we hypothesize a two-step process in the development and differentiation of the honey bee compound eyes. During the first step, which takes place from mid to end of pupation, the photoreceptor cell meets its fate and the corresponding opsin is expressed. In addition, the rhabdoms increase in length and diameter, and absolute opsin expression level rises. In a second step, which takes place after eclosion, when the determination of photoreceptors has already been completed, the quantity of opsin mRNA in both workers and drones increases drastically. This upregulation of opsin expression suggests an early adult maturation phase which takes place during the first days after eclosion, when workers and drones remain in the dark hive and only gradually come into contact with light during the transition from nurses to foragers or during their first orientation flights [37, 38]. This delayed transition from indoor to outdoor activities, when the visual system is in need, may have allowed the bees to postpone part of their eye development into the early adult phase in the course of evolution. Similar results have also been found in ants, where expression levels of all three opsins significantly increase during the first few days of adult life [39]. Such advancement in development might illustrate a common mechanism in eusocial insects that fulfill an age dependent division of labor, including a transition from indoor to outdoor tasks. A regulation of visual components dependent on age, light environment and circadian clock in honey bees has been discussed in an earlier study that found lower expression levels of the *Lop1* and *arrestin* mRNA in young in-hive bees compared to foragers [26]. Moreover, adult maturation has also been found in honey bees at neuronal and behavioral levels. Visual and olfactory boutons of projection neurons in the mushroom bodies significantly increase in their size between 1-day old nurse bees and foragers [40]. Also, honey bee mushroom body calyxes show a distinct volume increase during the first week after eclosion [41] and 1-day old bees have shown to be less phototactic than 7-day old bees [42]. In contrast to our results in honey bees, opsin mRNA levels in *Drosophila* are higher during the late pupal phase compared to adult flies [36], which would support our hypothesis that adult retinal (and neuronal) maturation is restricted to animals which undergo an age dependent transition from indoor to outdoor activities.

All our samples were collected at the same time of the day, since it has been shown that opsin expression in insects (Noctuidae: [24]) and vertebrates [25] might be regulated by the circadian clock to synchronize their endogenous rhythm with the environment. In flies and ants, for example, electroretinography of the compound eyes revealed a daily cycling of light sensitivity under constant darkness [43, 44]. Therefore, we investigated whether, in addition to the discovered developmental changes, opsin expression also possesses diurnal plasticity in adult bee eyes. Expression levels of all opsins were found to vary significantly during 24 h even under constant darkness, suggesting that their expression is under control of the endogenous clock (Fig. 3). Similar expression patterns have been shown for the *Lop1* gene by means of qPCR and northern blot analyses [26, 45]. We found highest expression levels during late night and putative early morning for all opsins, matching the regular activity pattern of foraging bees. Foragers usually show a distinct locomotor activity during the day [46] and rest during the night [47], matching the necessity of a well-equipped and sensitive visual system for diurnal activities outside the hive. Furthermore, the activity peak also correlates with highest availability of pollen and nectar during the morning [48]. As previously stated, the processing of visual components, such as opsins, in the honey bee seems to be regulated and influenced by a variety of factors e.g. retinal development, age, light environment, availability of food sources and the endogenous clock.

## Conclusions

Retinal ontogeny differs between honey bee workers and drones. Our results clearly show a sex-specific and spectral type-specific onset of opsin expression, and further that opsin mRNA expression levels evidently coincide with rhabdom and photoreceptor development during the pupal phase in honey bees. Since opsin expression levels still increase in young bees, we hypothesize that the elongated retinal maturation during the adult phase became possible due to the delayed transition from indoor to outdoor activities in bees. Future studies comparing the retinal opsin expression pattern of social insect species characterized by an age polyethism (e.g. honey bees) with those exhibiting alloethism (e.g. bumble bees) will allow to verify this hypothesis. In summary, our study provides a first basis for future studies aiming to unravel the proximate mechanisms resulting in a retinal mosaic of the honey bee compound eye.

## Methods

### Bee keeping and collecting

Honeycombs containing brood of *Apis mellifera carnica* were transferred from hives, located at the bee facility of the University of Würzburg, to the laboratory and were kept in an incubator at 34 °C and ~ 60% humidity at constant darkness. We identified eight pupal stages in workers (P1-P8; Fig. 1a) and nine stages in drones (P1-P9; Fig. 1b), following the classification of A Tofìlski [49], and V Dietermann, JD Ellis and P Neumann [50]. We collected at least 15 pupae from each stage at always the same time of the day (12 am – 2 pm) to account for possible diurnal variation of opsin expression (see below). Additionally, we collected newly emerged workers and drones (NE, 0-24 h old), as well as 7-day (A7) and 14-day old bees (A14, workers only) and kept them under constant darkness. For this, newly emerged individuals were transferred to small wooden cages and provided with Apiinvert (a liquid bee food containing pure sucrose; Südzucker, Mannheim, Germany) and a mixture of pollen and honey until they reached the respective age.

To evaluate if opsin expression in adult bees changes during the day and if it is under the control of the circadian clock, newly emerged workers were entrained to a light-dark rhythm (8:00 h – 20:00 h, CET) for 13 days and were kept in constant darkness on day 14. From these bees, individuals were collected at four-hour intervals over a time span of 24 h at day 14, adding up to six individuals per sampled time point (CT2, CT6, CT10, CT14, CT18, CT22). All collected pupae and adolescent bees were immediately transferred to liquid nitrogen and stored at − 80 °C until further processing.

### Dissection, RNA extraction and cDNA synthesis

We removed both eyes (the retina and a small part of the optic lobe) of workers since the different photoreceptor types seem to be approximately evenly distributed in the compound eye [21]. In contrast, drones lack green photoreceptor cells in the dorsal part of their eye [17, 22, 23], and we thus separated the dorsal from the ventral part. However, the ventral worker-like part of the retina covers only ca. 20% of the entire eye [22, 23], and we were unable to clearly dissect this part of the eye without contamination and thus used only the dorsal part in drones for further analysis. The eyes were dissected on ice and remained frozen until they were transferred into PCR tubes for further RNA extraction. To isolate mRNA, we used TRIzol Reagent (5 Prime, Hilden, Germany) and the Gold HP total RNA Kit (Peqlab Biotechnologie, Erlangen, Germany) following the manufacturer’s protocol. Quantity and quality of mRNA was estimated by spectrophotometric measurement and gel-electrophorese (1% agarose gel, stained with Midori Green Direct; Nippon Genetics Europe, Dueren, Germany). cDNA synthesis was performed by means of QuantiTec Reverse Transcription Kit (Quiagen, Hilden, Germany) according to the manufacturer’s protocol. All cDNAs were stored at -20C° until used for quantitative real-time PCR (qPCR).

### Opsin primers and qPCR

We used qPCR to evaluate specific mRNA expression levels of the honey bee opsins. Four different visual opsin genes have been identified in the honey bee so far: UV-sensitive opsin, *UVop*, blue-sensitive opsin, *BLop*, [18] and two long-wavelength sensitive opsins, *Lop1* and *Lop2* [19, 23, 51]. Since it has been reported that *Lop2* expression is restricted to the ocelli [23], we focused on the expression of *Lop1* in the honey bee compound eye. As an internal control we used the *Ribosomal protein 49* (*Rp49*) which has been previously shown to be a stable housekeeping gene in *Apis mellifera* [52]*.* Amplification efficiency for each primer pair was determined by means of a serial 1:10 dilution ranging from 10^1^ to 10^6^ copies (Table 1).

**Table 1:** Primers for qPCR

Gene	Forward primer	Reverse primer	Product length in bp	Primer efficiency
*UVop*	*5’-TAACTGGAATCGGTGCTGCG-3′*	*5’-CCCCATACTCCCATCACAGG-3′*	172 bp	0.97
*BLop*	*5’-AAGACTCTCGCCGGTAAAGC-3′*	*5’-GATGATCGCGAGTCCGATGT-3′*	174 bp	0.97
*Lop1*	*5’-CAAAAAGTCTTCGCACGCCA-3′*	*5’-AGCCACATCCGAACAAGGAG-3′*	177 bp	0.96
*Rp49*	*5’-CGTCATATGTTGCCAACTGGT-3′*	*5’-TTGAGCACGTTGAACAATGG-3′*	150 bp	0.91

Primers were designed based on the reported sequences of the three opsin genes [18, 19, 51] using PrimerBLAST (NCBI, Bethesda, USA) and ordered from Metabion (Planegg, Germany) (Table 1). All qPCR runs were performed with a Mastercycler ep realplex (Eppendorf, Hamburg, Germany). For all qPCR reactions we used the Kapa SYBR Fast qPCR Kit (Peqlab Biotechnologie) and prepared each qPCR master mix according to the manufacturer’s protocol. Each reaction was performed in a total volume of 20 μl, and PCR conditions for all reactions were as the following: 95 °C for 5 min once, followed by thermal cycling at 95 °C for 2 min, 3 s at 95 °C, 20 s at 65 °C and 8 s at 72 °C for 35 cycles, and finally completed with the execution of a melting curve to test for primer specificity. Furthermore, experimental conditions were evaluated in technical triplicates for each sample.

### Histological labeling of the pupal retina

Pupal retinae were dissected and fixed overnight at 4 °C in 4% formaldehyde in PBS. Fixed retinae were rinsed 3 times in PBS for 10 min and subsequently embedded in 5% low-melting-point agarose. We performed 80 μm sections by means of a vibrating microtome (Leica VT 1000S, Wetzlar, Germany) and rinsed them once for 10 min in PBS with 2% Triton-X 100 and twice in PBS with 0.2% Triton-X 100. Sections were then incubated in Alexa Fluor 488 Phalloidin (2.5 μl Phalloidin from methanol stock solution in 500 μl PBS; Molecular Probes, Eugene, Oregon, USA) for 1 day at 4 °C to label f-actin. Sections were rinsed three times in PBS and subsequently incubated in Hoechst 34580 (1:1000; Life Technologies GmbH, Darmstadt, Germany) for 15 min at room temperature to label cell nuclei. Finally, sections were rinsed in PBS five times, transferred to 60% Glycerol in PBS for 30 min at room temperature and mounted in 80% glycerol in PBS on slides. To visualize the retinae, we used a confocal laser-scanning microscope (Leica TCS SP2 AOBS, Leica Microsystems AG, Wetzlar, Germany) equipped with an argon/krypton and a three diode laser. Single images of sections were taken at a resolution of 1024 × 1024 pixels with a HC PL APO objective lens: 20/0.7 NA imm; with additional digital zoom: 2.0-4.0. Furthermore, scanned images were processed and edited in ImageJ 1.51e (Wayne Rasband National Institutes of Health, Maryland, USA) and CorelDRAW Graphics Suite X7 (Corel Corporation, Ottawa, Ontario, Canada).

### Experimental and statistical analysis

To evaluate the relative opsin expression, we used the 2^- ΔΔCT^- method [53, 54]. Opsin expression data of the pupal development of workers and drones as well as opsin data from adolescent development were normalized separately for each opsin gene to the housekeeping gene *Rp49* and the expression level of newly emerged bees (NE; set to one). We calculated and estimated the relative proportions of opsin gene expression as followed:

$$ \frac{OE_{gene}}{OE_{all\  genes}}=\frac{1/{\left(1+{E}_{gene}\right)}^{CT_{gene}}}{\sum 1/{\left(1+{E}_{gene}\right)}^{CT_{gene}}} $$

Whereby OE_gene_ is defined as the expression of each individual opsin gene and E_gene_ as the primer efficiency for the respective gene. CT_gene_ depicts the mean threshold cycle determined during qPCR for each pupal stage (for more details see: [55]). Expression data of the circadian expression experiment were treated as described above, and the time point of the lowest expression level of each gene was set to one. We used a general linear model (GLM) with Poisson’s distribution to analyze differences in opsin expression during pupal development and between sexes. Expression levels at different circadian time points were evaluated by means of log-linear one-way ANOVA. A Kruskal-Wallis test was applied to evaluate possible differences in rhabdom length and diameter during pupal development of workers. All statistical analyses were performed in R software v. 3.3.1.
